# Gallbladder Perforation Secondary to Enteric Fever: An Interesting Case of Acute Abdomen

**DOI:** 10.7759/cureus.4516

**Published:** 2019-04-22

**Authors:** Mustafa N Malik, Tayyab Mahmood, Asim Tameez Ud Din, Shehroz Aslam, Maria Imtiaz

**Affiliations:** 1 Internal Medicine, The University of Arizona, Tucson, USA; 2 Internal Medicine, Federal Government Services Hospital, Rawalpindi, PAK; 3 Internal Medicine, Rawalpindi Medical University, Rawalpindi, PAK; 4 Internal Medicine, Maricopa Medical Center, Phoenix, USA; 5 General Surgery, Jinnah Hospital, Lahore, PAK

**Keywords:** enteric fever, perforation, gallbladder, cholecystitis, cholecystectomy

## Abstract

Enteric fever is a common infectious disease, especially in countries with poor sanitation and in the tropics. It is caused mainly by Salmonella typhi and accounts for nearly 27 million cases worldwide and 200,000 deaths annually. Enteric fever involves the reticuloendothelial system such as bone marrow, spleen, and liver. As it mostly involves the Peyer’s patches of the terminal ileum, enteric perforation occurs commonly. However, gallbladder perforation can also occur, though not very often. Ultrasound as well as computerized tomography (CT) abdomen and pelvis lack specificity for detecting gallbladder perforations in enteric fever. Diagnosis is usually confirmed intraoperatively when the gallbladder is visualized and perforation is seen. Gallbladder perforation is usually seen in acute cholecystitis when the gallbladder becomes necrotic and gangrenous. In acalculous cholecystitis, perforation is rare. Enteric fever is one of the rarest causes of acalculous cholecystitis, leading to perforation. Here, we present the case of a 20-year-old man who presented with fever for 10 days along with loose stools, vomiting, and acute abdomen. Labs showed leukopenia, positive Typhidot test but X-ray erect abdomen and ultrasound abdomen and pelvis were nonspecific. Only after resuscitation and exploration of the abdomen was it found that the gallbladder had multiple perforations. The patient was improved after eight days of postoperative intravenous antibiotics. This is a unique and rare presentation of such a common infectious disease.

## Introduction

In many developing countries with little supply of clean drinking water and poor sanitation, typhoid fever is a common infection [1]. Already a rare entity, gallbladder perforation is extremely rare when it occurs as a complication of enteric fever [2]. The incidence of gallbladder perforation in enteric fever is around 3%-10% [3]. Salmonella, the causative agent of typhoid fever, has the ability to invade gallbladder epithelial cells, causing damage to the gallbladder wall, which then leads to perforation [4]. Pre-operative diagnosis is usually very difficult and has a high mortality rate [3]. We report the case of a 20-year old man with perforation of the gallbladder, who presented with an acute abdomen and improved after surgical intervention.

## Case presentation

**Figure 1:**
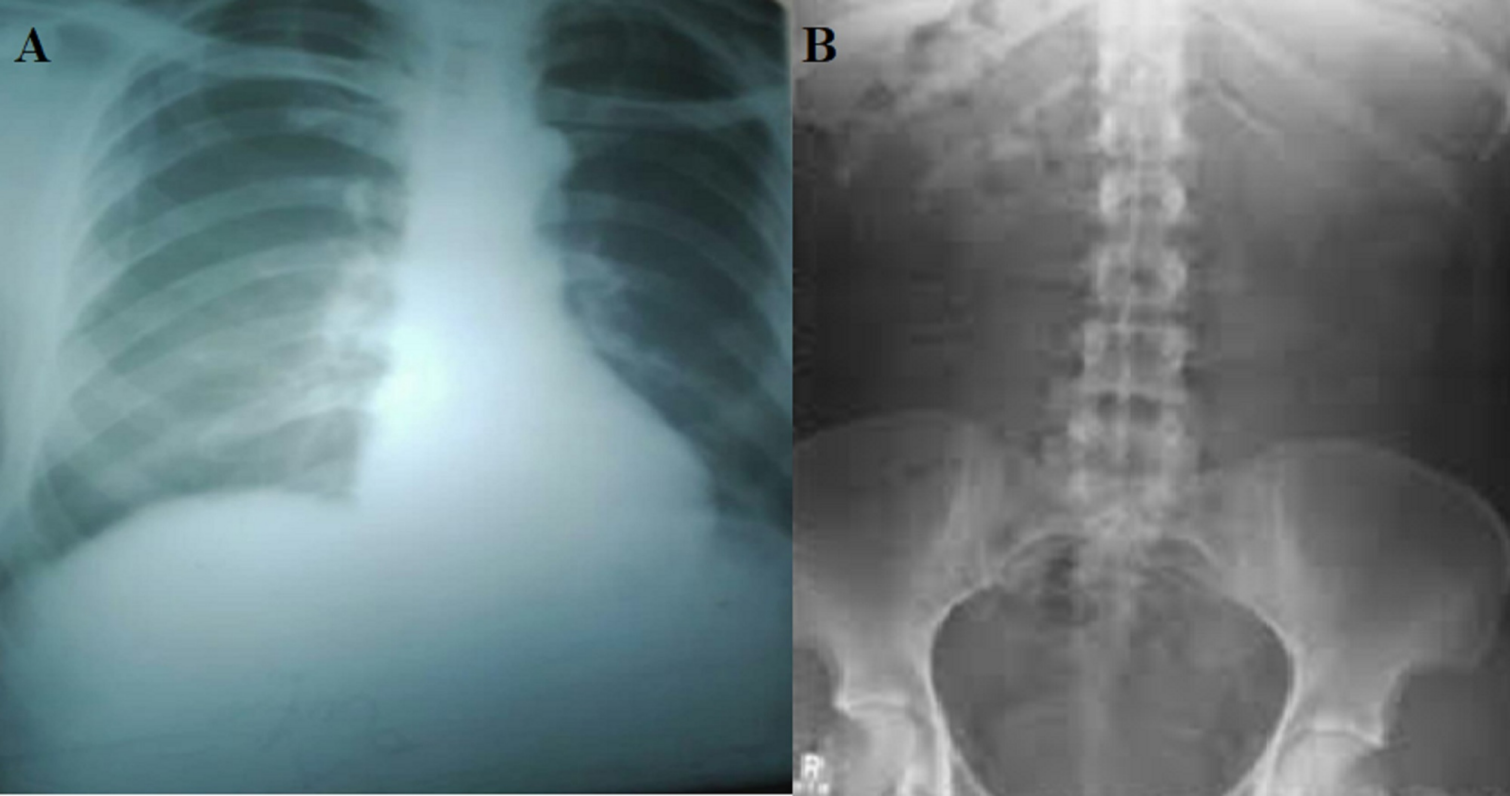
A) Chest X-ray showed no air under the diaphragm; B) Abdominal X-ray (erect) showed no gut distension

A 20-year-old male presented with a history of abdominal pain, diarrhea, and vomiting for six days along with fever for 10 days. The pain was gradual in onset, diffuse in nature, started in the epigastrium, migrated to the right hypochondrium, moderate in severity, aggravated by movement, and not completely relieved by analgesics. There were seven to eight episodes of loose stools per day, which were brown in color, soft in consistency, and often foul smelling. There were two to three episodes of vomiting per day; the vomitus was green in color, a cupful in quantity, often preceded by food intake. The fever was high grade, documented up to 103⁰ F, occurred 10 days before presentation, and followed a step-ladder pattern. On examination, there was visible pallor with tachycardia (pulse 115 beats/minute) and tachypnoea (respiratory rate of 18 breaths/minute); blood pressure was 110/60 mmHg; and temperature was 101⁰ F. On abdominal examination, the umbilicus was everted, the abdomen was distended, abdominal guarding was present with board-like rigidity, and maximum tenderness was at the right iliac fossa. Bowel sounds were sluggish, and digital rectal examination (DRE) was unremarkable. Hemoglobin was 11 g/dL, platelet count was 170,000 /µL, and white blood cell (WBC) count was 1650/µL (neutrophils 60%, lymphocytes 24%). Peripheral smear showed normocytic, normochromic red blood cells with severe leukopenia. Liver function test, renal function test, serum electrolytes, serum albumin, and coagulation profile were in the normal range. The typhoid test (both IgG and IgM) was highly positive for Salmonella typhi. Complete urine examination was unremarkable. Chest X-ray showed no air under the diaphragm and an erect abdominal radiograph showed no air fluid levels (Figure 1). Abdominal ultrasonography demonstrated minimal to mild abdominopelvic ascites.

**Figure 2:**
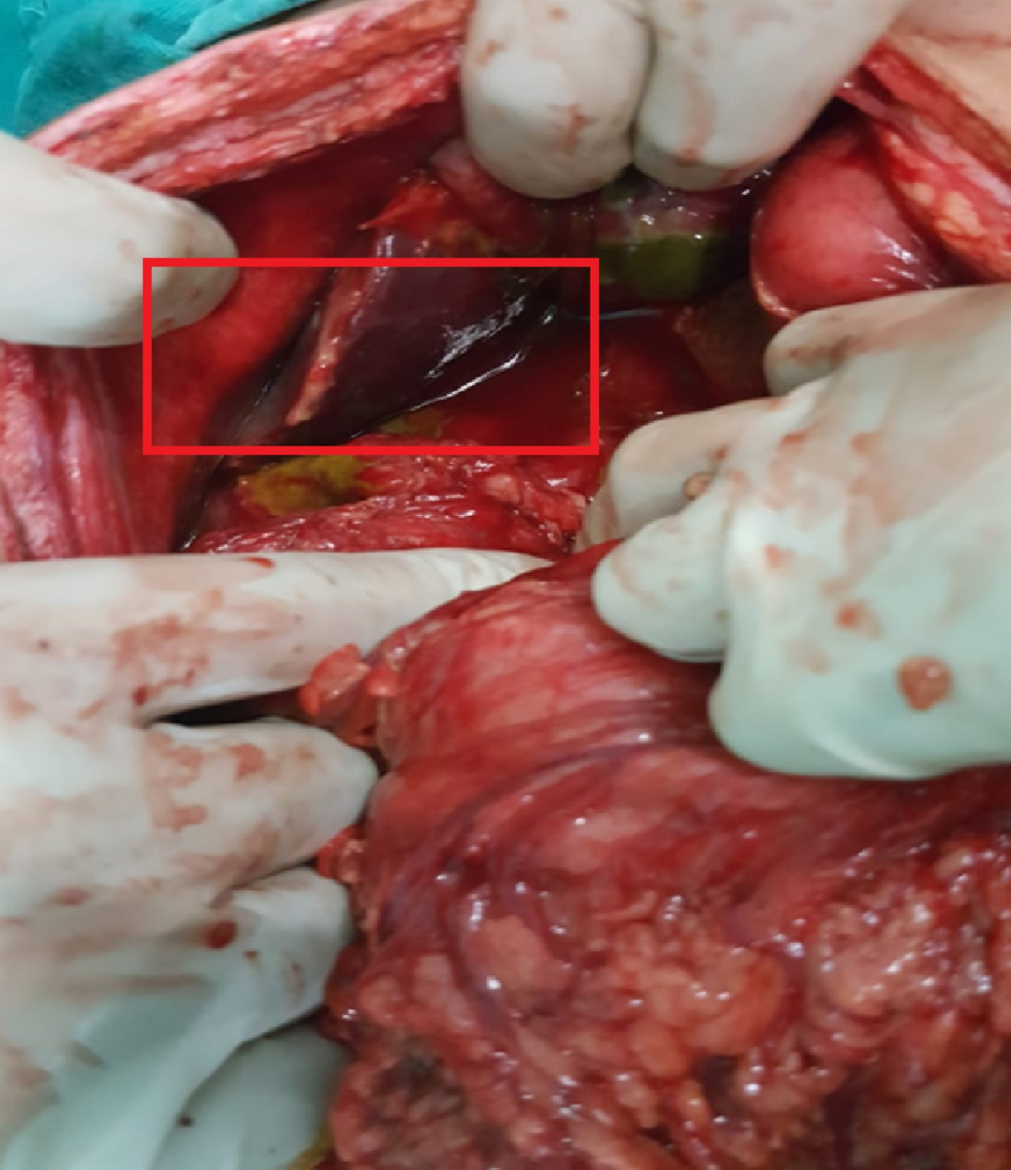
Intra-operative view of gallbladder perforation as a complication of enteric fever

After resuscitation, the patient was explored by a midline incision. The peritoneal cavity was containing 200 ml of bilious fluid and there were multiple flakes on the bowel loops. On further exploration, there were multiple perforations in the gallbladder of variable sizes (approximately 1-2 cm in size) (Figure 2).

No gall stones in the gall bladder, cystic duct, common bile duct, or peritoneal cavity were found. Cholecystectomy was done and the pelvic drain was placed after thorough peritoneal lavage. Postoperatively, intravenous ceftriaxone and gentamicin were administered for one week. There was uneventful recovery, and the patient was discharged on the eighth postoperative day.

## Discussion

Gallbladder perforation is an uncommon but potentially life-threatening condition. One of the most common causes of gallbladder perforation is acute calculous cholecystitis. The most common site is the distal part of the gallbladder due to its blood supply. Although rare, it can also occur in acalculous cholecystitis [5]. Less common causes include perforation secondary to Ascaris lumbricoides infection of the biliary tract, trauma, tuberculosis, and typhoid/enteric fever [6-7]. Niemeier classified gallbladder perforation into three types (Table 1) [8-9].

**Table 1:** Types of gallbladder perforation according to Niemeier’s classification

Type	Onset	Explanation [8, 9]
Type 1	Acute	Involves the perforation of the gallbladder into the peritoneal cavity; it is not surrounded by any protective adhesions
Type 2	Sub-acute	Consists of a perforated gallbladder surrounded by an abscess that is walled off by adhesions
Type 3	Chronic	Includes the formation of a fistula between the gallbladder and some other viscera

Enteric fever is caused by Salmonella typhi or Salmonella paratyphi A and accounts for nearly 200,000 deaths worldwide [3]. It is an acute illness characterized by fever, anorexia, nausea, vomiting, sometimes constipation, and bloody diarrhea along with hepatosplenomegaly and pancytopenia [10]. It involves the reticuloendothelial system, which includes the bone marrow (10-fold higher bacterial count as compared to that in the blood), liver, and spleen [3]. Pancytopenia occurs due to phagocytosis of red blood cells, platelets, and leukocytes in bone marrow [3]. Typhoid fever usually affects the terminal ileum and, rarely, the jejunum or cecum, causing perforation [11]. Salmonella concentrations in the epithelial cells of the gallbladder can exceed those in the liver and spleen, which can lead to perforation as well [4]. There is a high mortality rate in gallbladder perforation (around 12%-16%) as compared to the intestinal perforation in enteric fever [3]. Gali BM et al. reported two cases of gallbladder perforation complicating typhoid fever in boys aged 13 and 16 years [2]. In our case, it was a young man who presented with fever for 10 days and a rigid, boardlike abdomen along with leukopenia and positive Typhidot test, pointing towards a diagnosis of enteric fever with an acute abdomen. Gallbladder perforation usually occurs in elderly patients with acute cholecystitis [12]. In young patients, gallbladder perforation is rare and occurs as a result of intense inflammation [12]. This appears to have occurred most likely in our case, as the patient was young, with no clinical or radiographic evidence of cholecystitis.

Ultrasound has a low specificity for diagnosing gallbladder perforation. Only 20%-30% of cases are diagnosed correctly prior to surgery [13-14]. CT scan of the abdomen proved superior to ultrasound in detecting gallbladder perforation, pericholecystic fluid, streaky omentum, or mesentery [15]. Cholecystectomy is the treatment of choice in gallbladder perforation [16]. In uncomplicated cases, antibiotics (fluoroquinolones or ceftriaxone 1-2 g/day) are given for five to seven days. In complicated cases, treatment is given for 14-21 days [3]. In our case, antibiotics (IV ceftriaxone and gentamicin) were given for seven days.

## Conclusions

Gallbladder perforation secondary to enteric fever requires a high degree of clinical suspicion. In a patient with signs and symptoms of typhoid fever, acute abdomen might be due to enteric or gallbladder perforation. The latter, although less common, should be excluded. Early diagnosis and intervention significantly reduce morbidity and mortality. Cholecystectomy is the treatment of choice.
